# A rare non-canonical splice site in *Trema orientalis SYMRK* does not affect its dual symbiotic functioning in endomycorrhiza and rhizobium nodulation

**DOI:** 10.1186/s12870-023-04594-0

**Published:** 2023-11-24

**Authors:** Sultan Alhusayni, Yuda Purwana Roswanjaya, Luuk Rutten, Rik Huisman, Simon Bertram, Trupti Sharma, Michael Schon, Wouter Kohlen, Joël Klein, Rene Geurts

**Affiliations:** 1 Laboratory of Molecular Biology, Cluster of Plant Development, Plant Science Group, Wageningen University, Droevendaalsesteeg 1, 6708 PB Wageningen, The Netherlands; 2 Biological Sciences Department, College of Science, King Faisal University, 31982, Al-Ahsa, Saudi Arabia; 3 Research Centre for Applied Microbiology, National Research and Innovation Agency (BRIN), Cibinong, 16911 Indonesia

**Keywords:** Non-canonical splice site, SYMRK, LRR-type transmembrane receptor kinase, Mutualistic endosymbiosis, Nitrogen-fixing nodulation symbiosis, Arbuscular mycorrhizal symbiosis, *Parasponia andersonii*, *Trema orientalis*, Common symbiosis signalling pathway, Plant evolution

## Abstract

**Background:**

Nitrogen-fixing nodules occur in ten related taxonomic lineages interspersed with lineages of non-nodulating plant species. Nodules result from an endosymbiosis between plants and diazotrophic bacteria; rhizobia in the case of legumes and *Parasponia* and *Frankia* in the case of actinorhizal species. Nodulating plants share a conserved set of symbiosis genes, whereas related non-nodulating sister species show pseudogenization of several key nodulation-specific genes. Signalling and cellular mechanisms critical for nodulation have been co-opted from the more ancient plant-fungal arbuscular endomycorrhizal symbiosis. Studies in legumes and actinorhizal plants uncovered a key component in symbiotic signalling, the LRR-type SYMBIOSIS RECEPTOR KINASE (*SYMRK*). *SYMRK* is essential for nodulation and arbuscular endomycorrhizal symbiosis. To our surprise, however, despite its arbuscular endomycorrhizal symbiosis capacities, we observed a seemingly critical mutation in a donor splice site in the *SYMRK* gene of *Trema orientalis,* the non-nodulating sister species of *Parasponia*. This led us to investigate the symbiotic functioning of *SYMRK* in the *Trema-Parasponia* lineage and to address the question of to what extent a single nucleotide polymorphism in a donor splice site affects the symbiotic functioning of *SYMRK*.

**Results:**

We show that *SYMRK* is essential for nodulation and endomycorrhization in *Parasponia andersonii*. Subsequently, it is revealed that the 5’-intron donor splice site of *SYMRK* intron 12 is variable and, in most dicotyledon species, doesn’t contain the canonical dinucleotide ‘GT’ signature but the much less common motif ‘GC’. Strikingly, in *T. orientalis,* this motif is converted into a rare non-canonical 5’-intron donor splice site ‘GA’. This *SYMRK* allele, however, is fully functional and spreads in the *T. orientalis* population of Malaysian Borneo. A further investigation into the occurrence of the non-canonical GA-AG splice sites confirmed that these are extremely rare.

**Conclusion:**

*SYMRK* functioning is highly conserved in legumes, actinorhizal plants, and *Parasponia*. The gene possesses a non-common 5’-intron GC donor splice site in intron 12, which is converted into a GA in *T. orientalis* accessions of Malaysian Borneo. The discovery of this functional GA-AG splice site in *SYMRK* highlights a gap in our understanding of splice donor sites.

**Supplementary Information:**

The online version contains supplementary material available at 10.1186/s12870-023-04594-0.

## Background

Plants have evolved a range of mutualistic endosymbiotic partnerships with microbes to enhance nutrient uptake. The most ancient mutualistic endosymbiosis is the interaction between plant roots and Glomeromycota fungi, also known as arbuscular mycorrhizal (AM) fungi, which evolved over 400 million years ago [1]. Even today, AM endosymbiosis still occurs in ~ 72% of all higher plants [2]. Besides AM symbiosis, several plant lineages evolved additional or even alternative mutualistic endosymbiotic interactions, like orchid mycorrhiza, ericoid mycorrhiza, and diazotrophic rhizobia or *Frankia* bacteria hosted in root nodules. Interestingly, the evolution of these mutualistic endosymbiotic partnerships co-opted a signalling pathway critical for AM symbiosis. This pathway, known as the common symbiosis signalling pathway, is highly conserved and can be found in angiosperms, gymnosperms, monilophytes, and bryophytes [3].

The common symbiosis signalling pathway was first discovered in pea (*Pisum sativum*), showing to be critical for AM symbiosis and rhizobium-induced nodulation [4]. The subsequent molecular genetic characterisation in the legume models *Lotus japonicus* and *Medicago truncatula* revealed the pathway consists of four conserved components stretching from an LRR-type transmembrane receptor kinase down to the transcription factor *LjCYCLOPS/MtIPD3* [5, 6]. The LRR-type receptor kinase is generally named *SYMRK* (SYMBIOSIS SIGNALLING RECEPTOR KINASE), except for pea, *M. truncatula*, and *Medicago sativa,* where it is named PsSYM19, MtDMI2, and MsNORK, respectively [7]. The *SYMRK* extracellular structure varies between species, but in case of eudicots possesses a malectin domain, a conserved GDPC motif, and 2–3 LRR domains linked to a canonical intracellular serine-threonine kinase domain [7–10]. The malectin domain is cleaved in the absence of symbiotic signalling [11, 12]. Studies in *L. japonicus* showed that the remaining part of the SYMRK protein interacts with the LysM-type transmembrane receptor LjNFR5 [11, 12]. LjNFR5 is part of the receptor complex essential for recognising rhizobium-secreted lipo-chitooligosaccharide (LCO) signal molecules [13, 14]. Legume *symrk* knockout mutants are blocked in rhizobium LCO-induced signalling through the common symbiosis signalling pathway. Subsequently, nodule formation is not initiated, nor is *Rhizobium* infection initiated in *symrk* mutants [7, 8, 15, 16]. *Lj*SYMRK also interacts with the innate immune receptor LjBAK1 (BRASSINOSTEROID INSENSITIVE 1-ASSOCIATED RECEPTOR KINASE 1), which may allow repression of immune responses upon symbiotic infection [17]. Such a role is supported by *symrk* mutant analysis, revealing fortification of the plant cell wall upon infection with *Glomus mosseae* AM fungus or rhizobium in mutant or RNAinterference (RNAi) lines [18, 19].

Studies on *SYMRK* in non-legumes are limited. RNAi Knockdown studies in the actinorhizal plants *Datisca glomerata* and *Casuarina glauca* showed that, like in legumes, *SYMRK* is essential for nodulation [9, 20]. These findings demonstrate that the common symbiosis signalling pathway defines a conserved genetic basis for nodulation with rhizobia or *Frankia*.

More recent phylogenomic studies support the hypothesis that the nodulation trait has a single evolutionary origin in the last common ancestor of the orders Fabales, Fagales, Cucurbitales and Rosales, representing all ten nodulating plant lineages [20–22]. The occurrence of non-nodulating lineages in these four taxonomic orders allowed the identification of nodulation-specific genes, as such genes are prone to pseudogenization from the moment a plant lineage loses the nodulation trait. We identified seven of such nodulation-specific genes by comparing nodulating *Parasponia* species to their non-nodulating sister species of the genus *Trema* [23]*.* Among these is an *NFR5* orthologous LysM-type receptor named *NFP2,* essential for nodulation in *Parasponia* [24]. To our surprise, however, we also identified a seemingly critical mutation in *SYMRK* of *Trema orientalis* (accession RG33; *TorSYMRK*^*RG33*^), originating from the Sabah Provence in Malaysian Borneo [25]. It suggests that the *TorSYMRK*^*RG33*^ allele experiences pseudogenization, despite the fact *T. orientalis* accession RG33 can still establish an AM symbiosis [23].

*TorSYMRK*^*RG33*^ has a conserved gene structure, though has a mutation in the conserved dinucleotide motif in the 5’-donor splice site of intron 12, converting this generally highly conserved dinucleotide motif into ‘GA’. This led us to investigate the symbiotic functioning of *SYMRK* in the *Trema-Parasponia* lineage and investigate the impact of a seemingly critical SNP in an intron donor splice site in this gene.

## Results

### *Trema orientalis* and *Parasponia andersonii* differ in *Rhizophagus irregularis* colonization

Since *SYMRK* is known to be important for arbuscular mycorrhization in a range of species [7, 8, 20, 26], we first questioned whether *T. orientalis* accession RG33 can be effectively mycorrhized. To investigate this, we compared the mycorrhization dynamics of *T. orientalis* RG33 to *P. andersonii* (accession WU1). Both species are close relatives that diverged less than 20 million years ago [22], though have a somewhat different root architecture. *T. orientalis* plantlets have a shorter main root, whereas its lateral roots are longer when compared to *P. andersonii* (Fig. S1).

**Fig. 1:**
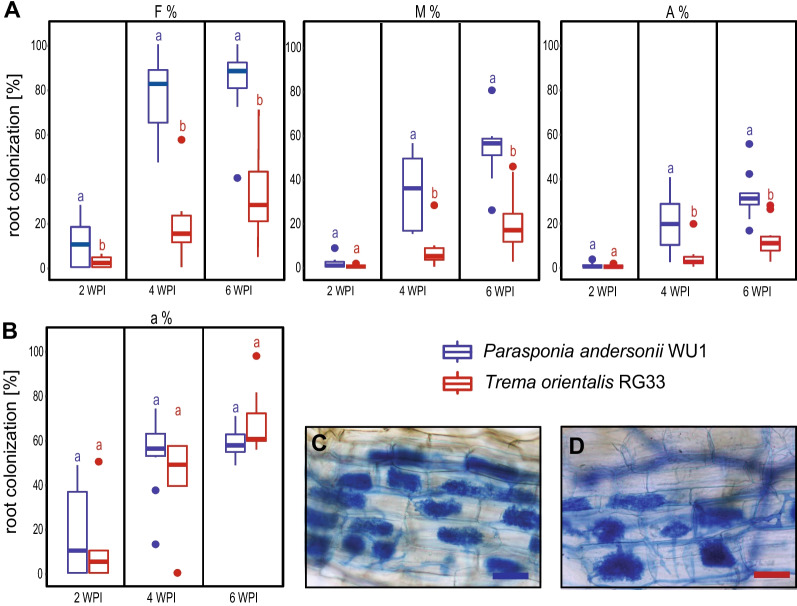
*Trema orientalis* accession RG33 and *Parasponia andersonii* accession WU1 differ in mycorrhizal colonisation. **A** Comparison of mycorrhization efficiency in the root system of *P. andersonii* WU1 (blue) and *T. orientalis* RG33 (red) at 2, 4 and 6 weeks post-inoculation with *Rhizophagus irregularis* DOAM197198. F%: The frequency of mycorrhiza in the root system. M%: the intensity of mycorrhizal colonisation in the root system. A%: Arbuscule abundance in the root system. **B** a%: Averaged arbuscule abundance detected in 50 randomly selected 1 cm infected segments of a root system. Error bars represent the SE of 10 biological replicates for each 50 × 1 cm root segment that has been analysed. Analysis was done according to Trouvelot et al. (1986) [26]. **C** Toluidine blue-stained *P. andersonii* and **D** *T. orientalis* root segment visualising *R. irregulates* arbuscules 6 weeks post-inoculation. Size bar = 10 μm

To compare the mycorrhization efficiency, seedlings of both species were inoculated with 125 spores of *Rhizophagus irregularis* DOAM197198. Mycorrhization was quantified for 6 weeks, focussing on the frequency of mycorrhizal presence in the root system(F%), the intensity of mycorrhization in the root system (M%), the arbuscule abundance in the root system (A%), and the averaged arbuscule abundance in randomly selected infected root segments (a%) [27]. This revealed a clear difference in mycorrhization colonization dynamics between both species. The root system of *P. andersonii* is broadly colonized, showing an abundant presence of hyphae 4 weeks post-inoculation (F% > 80%, M% > 50%, Fig. 1A, C). In contrast, *T. orientalis* RG33 showed a reduced mycorrhizal infection and a low abundance of mycorrhizal hyphae in the root (F% < 20%, M% < 10%, 4 weeks post-inoculation) (Fig. 1A, D). These reduced mycorrhizal infection rates of *T. orientalis* RG33 were also reflected in a reduced number of arbuscules found in the root system (A%). However, when evaluating the infected root segments, the arbuscule abundance (a%) was comparable to *P. andersonii* (Fig. 1B). This indicates that *T. orientalis* RG33 is infected less frequently by *R. irregularis* DOAM197198 when compared to *P. andersonii*. But once infected, the number of arbuscules formed in the infected root segment is similar between both species.

### *Parasponia andersonii SYMRK* is essential for arbuscular mycorrhization and nodulation

As *T. orientalis* RG33 can establish an arbuscular mycorrhizal symbiosis, we questioned whether *SYMRK* represents a single copy gene in the *Trema-Parasponi*a taxonomic lineage. We analysed genome sequences of 20 species representing monocots and major clades of dicots, including Fabales, Fagales, Cucurbiales, and Rosales species. The closest SYMRK paralogs of *P. andersonii* and *T. orientalis* were included as an outgroup. This revealed that *SYMRK* is a single-copy gene in the *Parasponia—Trema* lineage (Fig. S2).

Knock-down experiments in legumes and the actinorhizal species *C. glauca* and *D. glomerata* showed that *SYMRK* commits a dual role in establishing arbuscular mycorrhizal symbiosis and nodulation [7–9, 15, 16, 20, 28]. Furthermore, studies in *L. japonicus* revealed that ectopic expression of *LjSYMRK* results in the spontaneous onset of nodule organogenesis in absence of rhizobia [29]. To determine whether *SYMRK* in the *Parasponia-Trema* lineage fulfils a similar symbiotic role, two experiments were conducted. We generated CRISPR/Cas9 *symrk* knockout mutants in *P. andersonii* and conducted *PanSYMRK* ectopic expression studies in roots.

**Fig. 2:**
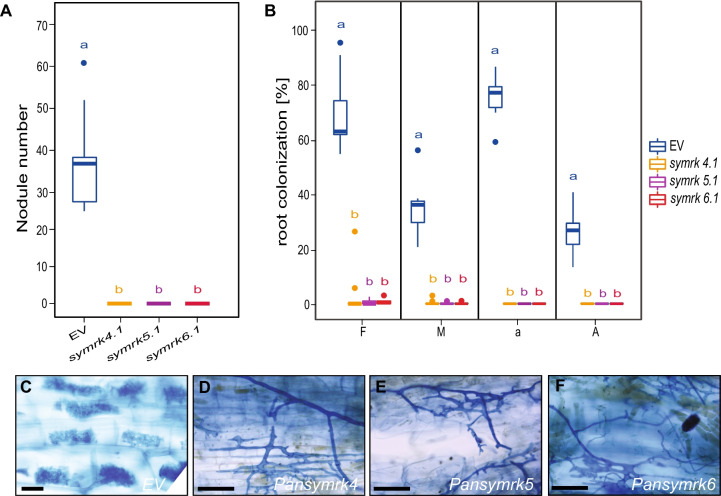
*Parasponia andersonii* SYMRK is essential for mycorrhization and nodulation. **A** Nodule numbers formed in *P. andersonii* empty vector control line (EV) and three *Pansymrk* mutant lines, 6 weeks post-inoculation with *Mesorhizobium plurifarium* BOR2. **B** mycorrhization efficiency in the root system of *P. andersonii* EV-control and three independent *Pansymrk* mutant lines 6 weeks post-inoculation with *Rhizophagus irregularis* DOAM197198. F%: The frequency of mycorrhiza in the infected root system. M%: the intensity of mycorrhizal colonisation in the infected root system. A%: Arbuscule abundance in the infected root system. a%: Averaged arbuscule abundance detected in 50 randomly selected 1 cm segments of a root system. Error bars represent the SE of 10 biological replicates, for each 50 × 1 cm root segment that has been analyzed. Analysis was done according to Trouvelot et al. (1986) [26] (C-F): Toluidine blue-stained *P. andersonii* EV-control **C**, *Pansymrk-4* **D**, *Pansymrk-5* **E**, and *Pansymrk-6* **F** root segment visualizing *R. irregulates* infections 6 weeks post-inoculation. Size bar = 10 μm

In total, three *Pansymrk* knockout mutant lines (homozygous line *Pansymrk-4* and the bi-allelic mutant lines *Pansymrk-5* and *Pansymrk-6*) were obtained by targeting the fourth and fifth coding exon using two single guide RNAs (sgRNAs) (Fig. S3A). All mutant alleles represent large deletions, only encoding a fragment of the extracellular domain (Fig. S3B). To determine whether *SYMRK* commits a key symbiotic function in *P. andersonii,* we first studied the nodulation phenotype of the *Pansymrk* mutants. *Pansymrk-4*, Pan*symrk-5* and Pan*symrk-6* plantlets were inoculated with *Mesohizobium plurifarium* BOR2, and the nodulation phenotypes were examined six weeks post-inoculation. The transgenic empty vector control plants (EV) were effectively nodulated, having nodule numbers ranging from 25 to 61 per plant. In contrast, the three *Pansymrk* mutant lines were unable to nodulate (Fig. 2A).

Next, we investigated the role of *PanSYMRK* in arbuscular mycorrhizal symbiosis. *Pansymrk-4, Pansymrk-5, Pansymrk-6*, and EV control plantlets were inoculated with an *R. irregularis* DAOM197198 spore suspension. Mycorrhization phenotypes were examined six weeks post-inoculation by quantifying four parameters; F%, M%, a%, and A%, as described above. The EV control plants interacted normally with the applied symbiont, with F%, M%, a%, and A% of 65,4%, 36,8%, 77,1%, and 26,1%, respectively (Fig. 2B, C). Although some intraradical hyphae were observed in a minority of the *Pansymrk* root segments (7 out of 417, 6 out of 760, and 9 out of 1085 segments) (Fig. 2B, D-F), generally, no arbuscules were observed in any of the tested *Pansymrk* mutant plantlets. This demonstrates that *SYMRK* is essential for nodulation and arbuscular mycorrhization of *P. andersonii* roots.

**Fig. 3:**
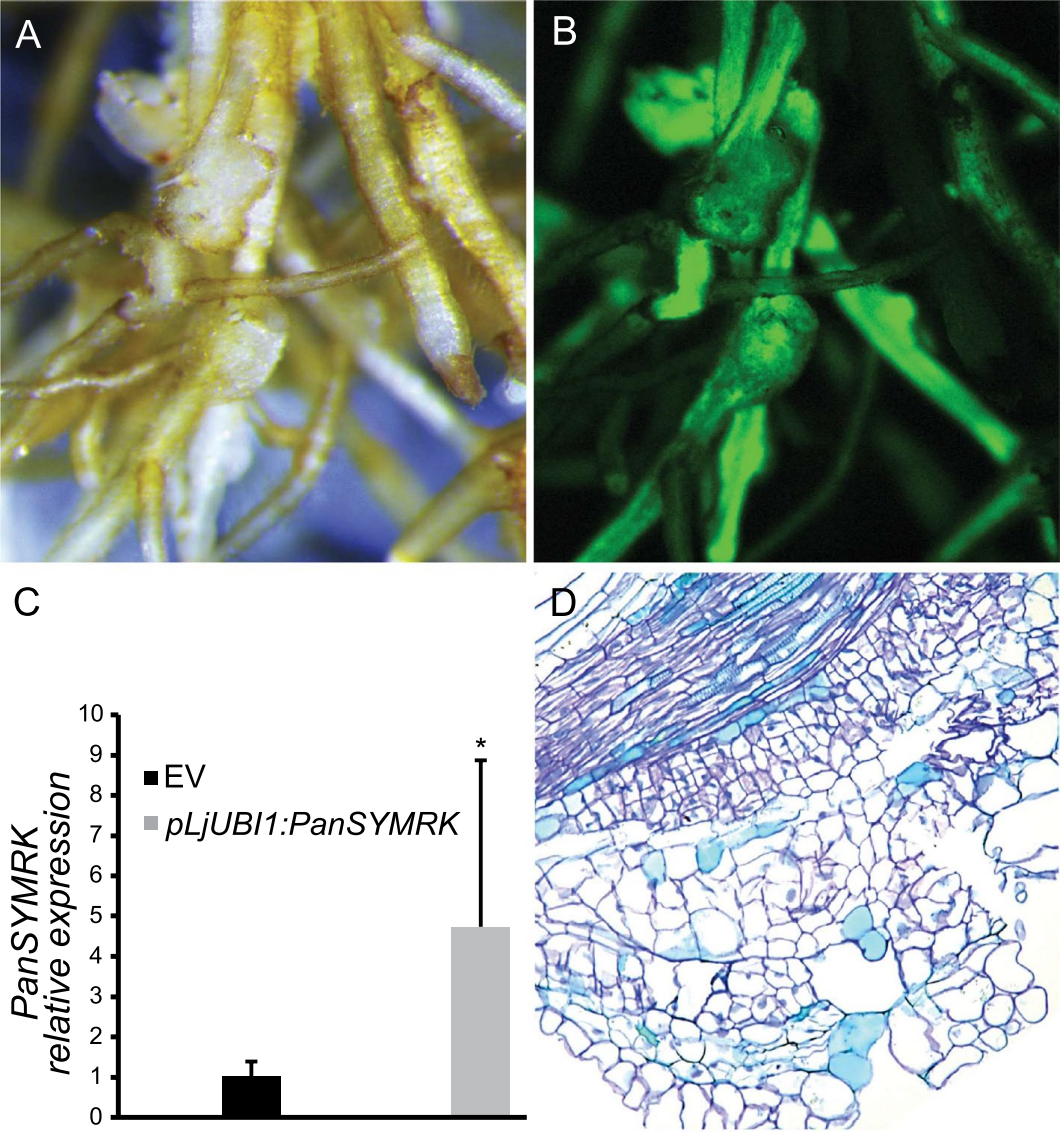
*PanSYMRK* ectopic expression induces spontaneous nodulation in *Parasponia andersonii*. (A, B) Bright-field **A** and green fluorescent image **B** of *P. andersonii* *A. rhizogenes*-transformed roots expressing GFP and *PanSYMRK* under control of the *pLjUBI1* promoter showing spontaneously formed nodule-like structures (6 weeks post planting). **C** Relative gene expression of *PanSYMRK* in *P. andersonii* *A. rhizogenes*-transformed roots containing an empty vector (EV) or *pLjUBI1:PanSYMRK* (*n* = 3). **D** Longitudinal section of a spontaneously formed nodule-like structure visualizing cortical and pericycle cell divisions

Next, we questioned whether the ectopic expression of *PanSYMRK* is sufficient for spontaneous formation of nodule-like structures. We employed *Agrobacterium rhizogenes*-mediated (*A. rhizogenes*) root transformation to introduce *PanSYMRK* driven by the *L. japonicus UBIQUITIN 1* (*LjUBI1*) promoter. This revealed spontaneous formation of nodule-like structures on roots ectopically expressing *PanSYMRK* (*n* = 5/25) (Fig. 3A-C). Longitudinal sections revealed that these nodule-like structures originate from dividing cortical and pericycle cells, similar to genuine *Parasponia* nodules (Fig. 3D). This led us to conclude that *SYMRK* is an essential key regulatory LRR-type receptor kinase for the onset of the nodule developmental program in the non-legume *P. andersonii.*

### The GA mutation of the 5’-donor splice site of intron 12 doesn’t affect *SYMRK *functionally

**Fig. 4:**
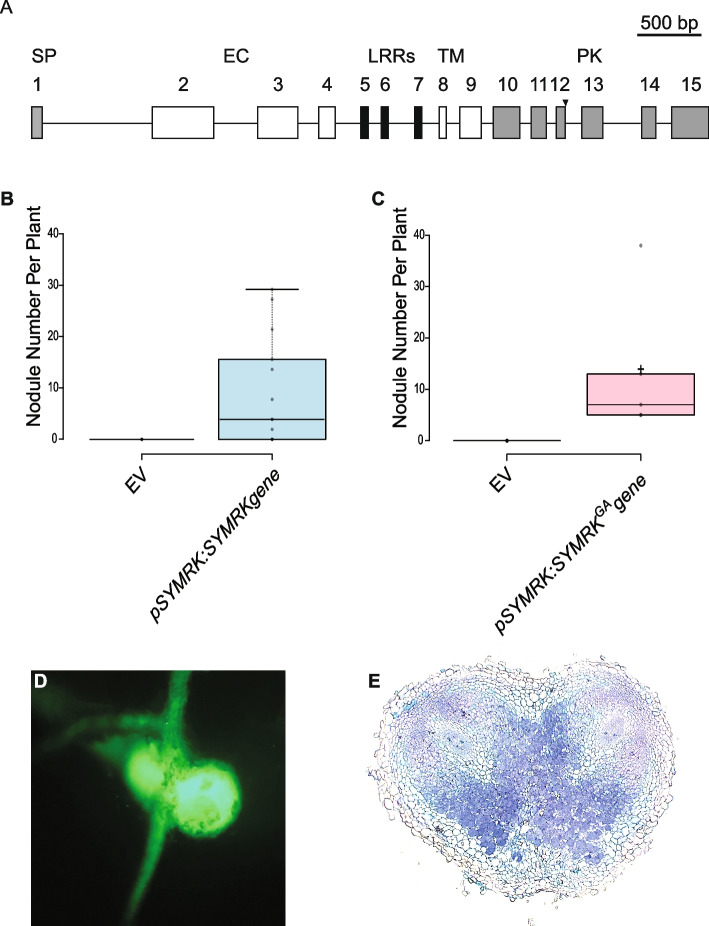
*Parasponia symrk-5* mutant trans-complementation of root nodule symbiosis. **A** Schematic representation of *P. andersonii* SYMRK gene structure. Arrowhead points to the location of the introduced GA mutation in *PanSYMRK* at the 5’-donor splice site of intron 12. **B** Nodule number per plant formed on *Pansymrk-5* *A. rhizogenes* transformed root with *pPanSYMRK:PanSYMRK* gene (*n* = 13). (C-E) Nodule number per plant (*n* = 5) **C**, representative image of green flurescence protein (GFP) nodule **D** and a section through a mature nodule **E** of *Pansymrk-5* *A. rhizogenes* transformed root with *pPanSYMRK:PanSYMRK*^GA^ carrying a GA mutation at the 5’-donor splice site of intron 12. Nodules were harvested and analysed at 8 weeks post inoculation with *Mesorhizobium plurifarium* BOR2 (OD600 = 0.025)

As *T. orientalis* RG33 -possessing a single *SYMRK* gene copy- can be mycorrhized effectively, it suggests that the *TorSYMRK*^*RG33*^ allele encodes a functional protein to support this plant-fungus symbiosis. Earlier studies in *M. truncatula* revealed that the *SYMRK* requirements differ between mycorrhizal colonization and rhizobium nodulation [7]. The *M. truncatula* R38 *dmi2* mutant possesses a missense mutation converting a glycine to glutamic acid mutation at position 794 of the protein [7]. This mutation affects the kinase phosphorylation activity and the capacity of the protein to interact with it’s downstream target 3-HYDROXY-3-METHYLGLUTARYL COENZYME A REDUCTASE1 (MtHMGR1) [30, 31]. *M. truncatula* R38 *dmi2* is affected in nodulation but not in mycorrhization, suggesting a SYMRK functional kinase domain is less critical for the latter interaction [7]. As the *T. orientalis SYMRK*^*RG33*^ may encode -at least in part- a truncated SYMRK protein lacking essential domains of the kinase motif (Fig. 4A), we question to what extent this allele could function in nodulation.

To investigate this, first the native promoter region of *P. andersonii SYMRK* was identified. We used *A. rhizogenes* root transformation to show that a ~ 3 kb upstream region including the 5’-UTR driving the *PanSYMRK* gene functionally complemented the *Pansymrk-*5 mutant (4.9 nodules/plant at 8 wpi) (Fig. 4B; Fig. S4B). Next, we used this promoter to drive a *PanSYMRK* gene mutant harbouring a GA at the donor site of intron 12, mimicking the *TorSYMRK*^*RG33*^ allele to determine its functionality in the *P. andersonii Pansymrk-*5 mutant background. Using *A. rhizogenes* root transformation, we found full complementation of the *Pansymrk* mutant phenotype (Fig. 4C-E; Fig. S4C). On average, 13 nodules per plant were formed at 8 wpi. Sections of these nodules revealed a wild type cytoarchitecture, including a large zone of cells possessing fixation threads. This shows that the GA point mutation at the donor site of intron 12 is not affecting *SYMRK* gene functionality.

### A GA 5’-donor splice site is very rare, though effectively spliced in *TorSYMRK*^*RG33*^

We question how effective an intron that possesses a GA as the first two nucleotides of a donor splice site is spliced. To determine this, we aimed to compare the coverage of RNAseq reads of the 15 exons and 14 introns of the *SYMRK* gene of *T. orientalis* and *P. andersonii*. *SYMRK* is highly similar in both species, though introns show some variation in length (Table 1). *SYMRK* is known to be expressed in the root [8]. We grew *T. orientalis* and *P. andersonii* seedlings in vitro on a low nitrate medium and subsequently isolated 1 cm regions of roots just above the root meristemic zone. RNA extracted from these samples was sequenced (in triplicates), mapped, and analysed (Fig. S5A). When focussing on intron 12, we found a per base mean coverage of 4.6 ± 1.0 for *TorSYMRK*^*RG33*^, whereas in *P. andersonii,* the coverage of this intron is only 0.2 ± 0.3 mean per base coverage (Table 1). Comparing the splice site efficiency of intron 12, we observe that GA splice site in *T. orientalis* splices efficiently at approximately 95%, while the GC splice site in *P. andersonnii* shows an efficiency of 99.9%. This difference in intron retention between *PanSYMRK* and *TorSYMRK*^*RG33*^ was also observed by qRT-PCR on root mRNA (Fig. S5B). These data suggest that *SYMRK* intron 12 is spliced less efficiently in *T. orientalis* when compared to *P. andersonii.* However, a similar variance is observed for other introns, which possess canonical donor and acceptor splice sites; e.g. *PanSYMRK* intron 11 (Table 1), suggesting some intron retention is not hampering gene function. Therefore, we conclude that SYMRK^RG33^ is fully functional, despite a non-canonical GA dinucleotide motif in the donor splice site.

**Table 1:** Splicing efficiency of *Trema orientalis* and *Parasponia andersonii SYMRK* in the root susceptible zone for symbiotic engagement

*SYMRK*	*T. orientalis*	root susceptible zone		*P. andersonii*	root susceptible zone	
	**length (bp)**	**coverage**	**s.d**	**length (bp)**	**coverage**	**s.d**
exon1	99	28.1	8.9	99	94.6	39.6
intron1	765	0.0	0.1	912	0.1	0.1
exon2	525	35.2	13.2	525	96.5	17.6
intron2	339	0.0	0.0	341	0.1	0.2
exon3	475	51.2	9.1	475	114.6	17.1
intron3	351	0.0	0.0	364	0.0	0.0
exon4	150	59.3	23.4	150	127.0	24.9
intron4	188	0.1	0.1	175	0.1	0.2
exon5	71	89.2	27.6	71	166.1	29.4
intron5	103	0.3	0.2	102	1.0	0.7
exon6	68	84.4	20.7	68	133.1	22.2
intron6	202	2.7	0.5	319	0.2	0.2
exon7	71	70.7	18.3	71	125.0	13.2
intron7	133	0.0	0.0	134	0.3	0.4
exon8	71	62.2	16.9	71	138.8	15.5
intron8	97	0.9	1.1	86	0.1	0.1
exon9	193	76.1	16.1	193	142.9	22.8
intron9	98	1.9	0.6	98	0.9	0.9
exon10	235	79.6	13.5	235	155.3	18.6
intron10	83	0.1	0.1	90	0.3	0.6
exon11	126	100.8	13.3	126	158.0	35.0
intron11	65	1.3	1.9	83	10.5	4.3
exon12	86	91.8	19.3	86	158.9	34.5
**intron12**	**118**	**4.6**	**1.0**	**118**	**0.2**	**0.3**
exon13	189	109.4	13.8	189	159.3	16.9
intron13	276	1.6	1.0	273	0.7	0.6
exon14	132	155.1	7.4	132	255.8	22.9
intron14	112	4.2	4.3	112	0.4	0.7
exon15	326	135.4	9.4	326	207.6	13.0

RNA-seq quantification for each intron and exon in the *SYMRK* gene of *Trema orientalis* and *Parasponia andersonii* is determined by the mean per base coverage of three biological replicates.

Next, we questioned how unique a GA donor splice site is in plants. For this, we analysed all annotated introns in *T. orientalis*, *P. andersonii,* and the model plant species *L. japonicus, M. truncatula*, and *Arabidopsis thaliana* [23, 32–34]. This showed that a GA donor splice site is extremely rare, varying from none in the annotated gene models of *M. truncatula* to 14 in *A. thaliana* (Table 2, Table S1).

**Table 2:** Frequency of predicted canonical and non-canonical donor splice sites. Splice site occurrences are based on existing gene models predictions for *Trema orientalis*, *Parasponia andersonii*, *Lotus japonicus*, *Medicago truncatula* and *Arabidopsis thaliana*

	***T. orientalis***		***P. andersonii***		***M.truncatula***		***L. japonicus***		***A. thaliana***	
**Splice motif**	**Total**	**%**	**Total**	**%**	**Total**	**%**	**Total**	**%**	**Total**	**%**
GT-AG	102,107	98.09%	100,852	98.36%	213,612	95.94%	148,570	97.99%	159,839	98.80%
GC-AG	537	0.52%	567	0.55%	8962	4.03%	2193	1.45%	1742	1.08%
AT-AC	13	0.01%	14	0.01%	79	0.04%	656	0.43%	90	0.06%
**GA-AG**	**11**	**0.01%**	**8**	**0.01%**	**0**	**0.00%**	**6**	**0.00%**	**14**	**0.01%**
Others	1426	1.37%	1096	1.07%	0	0.00%	190	0.13%	89	0.06%
Canonical	102,107	98.09%	100,852	98.36%	213,612	95.94%	148,570	97.99%	159,839	98.80%
Non-canonical	1987	1.91%	1685	1.64%	9041	4.06%	3045	2.01%	1935	1.20%
Grand Total	104,094	100.00%	102,537	100.00%	222,653	100.00%	151,615	100.00%	161,774	100.00%

### *Trema orientalis SYMRK*^*RG33*^ GA donor splice site is geographically limited

**Fig. 5:**
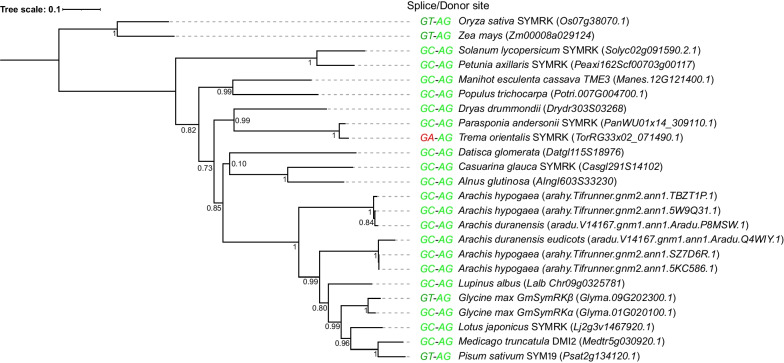
Phylogeny of SYMRK including the splice site dinucleotide motifs for intron 12. Phylogeny was reconstructed based on an alignment of SYMRK orthologous proteins from 19 species. Leaves are labelled by their respective species, gene name if available) and gene identifier. The non-canonical GC donor splice site is common in SYMRK intron 12 of dicotyledon species, except in *Glycine max* SYMRKβ and *Pisum sativum* SYM19, where GC is substituted by GT. In contrast, only *Trema orientalis* RG33 possesses a GA motif in this position (highlighted in red)

As *T. orientalis* RG33 possesses an extremely rare GA motif at the donor splice site of intron 12, we question to what extent such polymorphism is unique in *SYMRK*. First, we analysed SYMRK orthologs in a broad phylogenetic context. This showed that a non-canonical GC donor splice site is common in *SYMRK* intron 12 of dicotyledon species (Fig. 5).

**Fig. 6:**
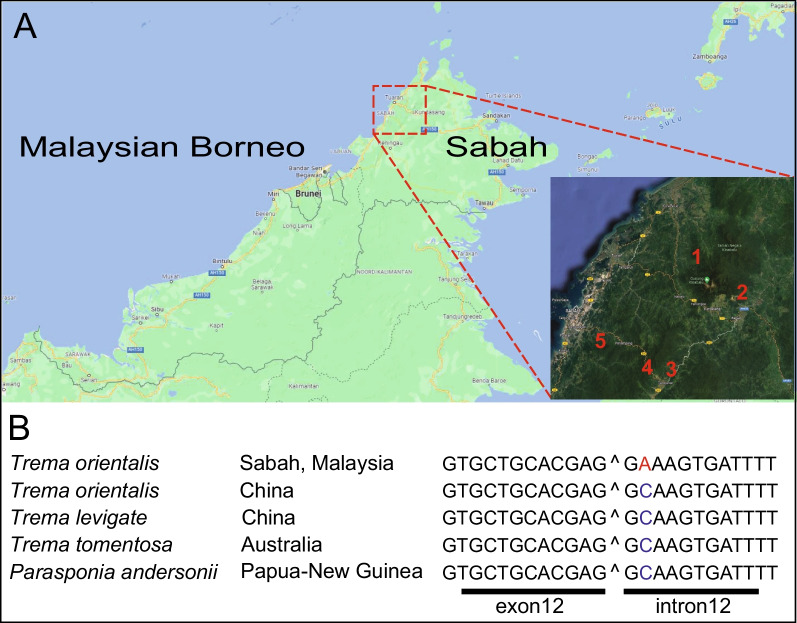
SYMRK intron 12 unique non-canonical donor splice site occurs in a *Trema orientalis* population endogenous to Sabah, Malaysia. **A** Locations of 28 *Trema orientalis* specimens collected in Malaysian Borneo, province of Sabah. 1: Sayap, 2: Poring, 3: Mahua, 4: Gunung Alab, and 5: Inobong. Plants were collected in 2012 as described in Merckx et al. (2015) [31] (see also Table S2). Map data © 2023 Google. **B** The ‘GA’ donor splice site of intron 12 is unique to *Trema orientalis* of Malaysia, Sabah, whereas related accessions and species possess a non-canonical ‘GC’ at this position in SYMRK

However, none of the analysed *SYMRK* genes possesses a GA motif at this position. Subsequently, we analysed *SYMRK* of the *Parasponia-Trema* species complex. Among others, *T. orientalis* accession RG33 was collected during an expedition in Sabah Provence, Malaysian Borneo, in 2012 [23, 25]. We analyzed 27 additional *T. orientalis* individuals collected from five distinct locations in Malaysian Borneo (Fig. 6A). All possess the rare GA intron 12 donor splice, whereas this mutation is absent in *Trema* and *Parasponia* accessions sampled outside Borneo (Fig. 6; Table S2). This demonstrates that the *SYMRK*^*RG3*^ allele is not unique, though it associates with the Borneo *T. orientalis* population.

## Discussion

The LRR-type receptor kinase *SYMRK* is a critical component in the common symbiosis signalling pathway controlling endosymbioses. In legumes, *SYMRK* is essential for rhizobium LCO-induced signalling. We identified a seemingly critical mutation of the conserved dinucleotide motif in the 5’-donor splice site in *T. orientalis SYMRK* accession RG33*. T. orientalis* is a non-nodulating relative of nitrogen-fixing *Parasponia* species and has experienced pseudogenization of several key nodulation genes [23]. Here we show that despite a mutation in a splice site motif, *TorSYMRK*^*RG33*^ remains a functional allele that can be effectively spliced. The dominant occurrence of the *TorSYMRK*^*RG33*^ allele in the Malaysian Borneo *T. orientalis* population underlines the splice site mutation is not affecting the fitness of the tree species.

Splicing is a highly conserved process in eukaryotes, requiring a spliceosome complex consisting of five small nuclear RNAs and several proteins. The vast majority of introns are spliced by the so-called U2-type spliceosome, recognizing two highly conserved di-nucleotide motifs at the start and end of the intron sequence, namely GT-AG. Bioinformatic studies in plant, animal, and fungal species indicate that alternative dinucleotide motifs are used in less than 2% of cases, among which GC-AG is the most abundant non-canonical splice motif representing 1.5% of all introns annotated in plant gene models [35, 36]. The GA-AG splicing motif, as found in *TorSYMRK*^*RG33*^ intron 12, is reported to occur in > 0.03% of the cases [36].

The mechanism driving the evolution of rare non-canonical splice sites remains elusive. The GA-AG dinucleotide splicing motif was found in higher frequency in two non-related animal species; the copepod *Eurytemora affinis* and the tunicate *Oikopleura dioica* [36–38]. However, it remains unknown whether both species have gained these by convergent evolution or, alternatively, it is an ancestral trait preserved in only a few species [36]. In the case of *SYMRK*, we noted that in related species, *SYMRK* intron 12 possesses the more common non-canonical GC-AG dinucleotide splice motif. This may lead to the hypothesis that such a GC-AG motif is the ancestral state allowing the evolution of the even more rare GA-AG motif. We inserted the GC to GA mutation in the *P. andersonii SYMRK* gene and showed that this variant is fully functional when expressed under its native promoter. This suggests that a simple single nucleotide polymorphism is sufficient to allow the evolution of the GA-AG dinucleotide splicing motif in *TorSYMRK*^*RG33*^. We analysed genomes of five plant species for gene models possessing a GA dinucleotide motif in the donor splice site. We found that the GA motif is indeed present in the annotated gene models, albeit at very low frequency in the analysed species.

Using CRISPR-Cas9 technology in *P. andersonii*, we demonstrated for the first time by mutant analysis that *SYMRK* commits a dual symbiotic role in essential nodulation and AM symbiosis in a non-legume. Earlier studies using RNAi in *C. glauca* and *D. glomerata* provided evidence that *SYMRK* is required for *Frankia*-induced nodulation and mycorrhization [9, 20]. *Parasponia*, *Casuarina*, and *Datisca,* together with legumes, represent all four taxonomic orders that contain nodulating species and for which *SYMRK* is an essential symbiotic gene. It supports the hypothesis that *SYMRK* -and other components of the common symbiosis signalling pathway- have been recruited to function in nodulation in a common ancestor that lived before the divergence of the Fabales, Fagales, Cucurbitales, and Rosales orders.

## Conclusions

This study of the LRR-type receptor kinase SYMRK in the non-nodulating relative of nitrogen-fixing *Parasponia* species, *T. orientalis*, led to the identification of a functional splice site mutation in the gene. The discovery of this rare non-canonical GA-AG splice site motif in *SYMRK* raises questions about the evolution of such motifs and the mechanisms driving their occurrence. Furthermore, this study demonstrates the conservation of SYMRK functioning in nodulation and AM symbiosis in both legumes and non-legumes. The *Parasponia-Trema* comparative system is established to obtain insight into the evolutionary trajectory of the nodulation trait. It uncovered several genes critical for rhizobium-induced nodulation in a non-legume [23, 24, 39]. Eventually, *Trema* species can serve as an experimental test system to uncover essential genes to rebuild the nodulation trait. Additionally, we demonstrated that the *Parasponia-Trema* comparative system is equally valuable to uncovering the functionality of rare non-canonical splicing motifs. Overall, this study contributes to our understanding of both the common symbiosis signalling pathway and the mechanisms of gene splicing in plants.

## Material and methods

### Plant materials and growth conditions

*Trema orientalis* plants used in this study were collected between September 10th and 25th, 2012, during the Crocker Range/Kinabalu Scientific Expedition. This expedition was conceived, organized, funded, and conducted jointly by Sabah Parks (Malaysia) and the Naturalis Biodiversity Center (The Netherlands). Detailed information about the expedition is available in Merckx et al*.,* 2015 [25]. Taxonomic analysis of the *T. orientalis* samples has been previously published in van Velzen et al*.,* 2018. [23]. *P. andersonii* WU1 and *T. orientalis* RG33 were grown and maintained as described previously [40, 41]. Plantlets for nodulation and mycorrhization assay were vegetatively propagated in vitro and rooted [40, 41].

### Mycorrhization assays and trypan blue staining

Mycorrhization assays were performed using a commercial spore of *Rhizopagus irregularis* (Agronutrion-DAOM197198, Carbonne, France). Spores inoculum, inoculation, and trypan blue staining were prepared and performed as described previously [41].

To quantify mycorrhization, a minimum of ~ 50 cm roots for each sample were cut into 1 cm fragments. 25–30 root fragments were placed on a single microscope slide, and 30% glycerol was added. Roots were covered with a cover glass and pressed until root fragments became flat. The frequency of mycorrhiza (%F), the intensity of mycorrhizal colonization (%M), and arbuscules abundance (%A) in the root system was scored and calculated according to Trouvelot et al. [27].

### Nodulation assay

*P. andersonii* plantlets for nodulation were inoculated with *Mesorhizobium plurifarium* BOR2 (OD600 = 0.05) [23, 40, 41]. Plants were removed from the pots six weeks post-inoculation, roots were washed with running water to remove perlite, and nodules were counted. In (*trans*) complementation studies, plant roots were examined under fluorescent stereo microscopy, and nodule number was quantified for each transgenic root (eight weeks post-inoculation with *Mesorhizobium plurifarium* BOR2 (OD600 = 0.025)).

### Root growth assay

Five seedlings of *P. andersonii* and *T. orientalis* RG33 were grown on ½ strength modified Hoagland medium in 12 cm square plates. Plants were grown vertically at a 60-degree angle for 21 days at 28 °C, 16/8 h day-night regime. The primary root was determined as the main root that emerged from cotyledon, whereas lateral roots were determined as roots that emerged from the primary root. Per plants, primary root length, the average number of lateral roots, and lateral root density (per cm main root) were determined 21 days post germination. Primary root growth was measured by following its development every day for 21 days post germination. The average lateral root length was determined by measuring its size in five selected lateral roots 21 days post germination.

### Vectors and constructs

Single-guide RNAs (sgRNAs) were designed using the ‘Find CRISPR Targets’ function implemented in Geneious software version 9.1.5 (Biomatters, New Zealand) and subsequently checked against the *P. andersonii* genome for high identity off-targets. For CRISPR/Cas9-mediated mutagenesis and complementation studies, binary transformation constructs were created using Golden Gate assembly as described previously [40, 41], and a list of constructs generated from both studies is listed in Table S3. For CRISPR/Cas9-mediated mutagenesis, two sgRNAs were used to target the fourth and the fifth coding exons of *PanSYMRK* (Fig. S3). Selected sgRNAs were amplified using sequence-specific forward primers and a universal reverse primer (Table S4), using Addgene plasmid no. 46966 as template [42]. To allow for Golden Gate cloning, BpiI and BsaI restriction sites in the putative promoter sequence of *PanSYMRK* were mutated by introducing single nucleotide substitution [43]. For the complementation study, the sequence of *P. andersonii SYMRK* promoter, 5’ untranslated region (5’ UTR), genomic DNA, 3’ untranslated region (3’ UTR), and terminator were synthesized. Also, a modified version of *P. andersonii SYMRK* genomic DNA was synthesized harbouring a point mutation at the donor splice site of the 12th intron, mimicking *T. orientalis SYMRK*^*RG33*^. (Invitrogen, Thermo Fisher Scientific, United States).

### Plant transformation

*Agrobacterium tumefaciens*-mediated transformation and genotyping were done based on previously published protocols [40, 41]. Primers used for genotyping are listed in Table S4. Hairy root transformations were performed according to Cao et al*.* [44], where *A. rhizogenes* MSU440 or AR1193 harbouring plasmid DNA of interest were used to infect micro-propagated plants wounded on their base. Infected plants were grown on agar plates of Schenk and Hildbrandt medium (SH medium) [45] and incubated at 21 °C for one week on a 16/8 h light/dark regime. Transformed plants were transferred to agar plates of SH medium supplemented with 10 g sucrose/L, cefotaxime 100 μg/mL, and kanamycin 50 μg/mL and subsequently incubated at 21 °C for one week followed by 28 °C for two weeks. Plants were checked for transgenic roots using a fluorescence stereo microscope.

### RNA Sequencing

For RNA isolation, tissue was harvested from a ~ 1 cm region just above the meristematic zone of young growing roots and snap-frozen in liquid nitrogen. Material from ~ 5 plants was combined to form a single biological replicate. RNA was isolated in triplicate as previously described [23]. Library preparation and RNA sequencing was conducted by BGI (Schenzhen, China). Mapped RNA-sequencing reads covering the *SYMRK* gene in *P. andersonii* and *T. orientalis* were visualized using Integrative Genomics Viewer (IGV) [46]. Based on the different splice sites, two *SYMRK* splice variants were manually constructed. Functional protein domains for these variants were annotated using InterProScan 5 [47].

### Phylogenetic reconstruction

Orthologs of SYMRK were identified among 49 publicly available proteomes by applying a Reciprocal Best Hits (RBH) approach, using *L. japonicus* SYMRK (Lj2g3v1467920.1) as the query sequence. Identified orthologous proteins were aligned using Clustal Omega 1.2.3. [48]. A phylogenetic SYMRK tree was constructed using PhyML 3.0 [49] with LG substitution model 1,000 bootstrap replicates and rooted on the two Poales outgroup species. The tree was visualized using the Interactive Tree Of Life (iTOL) tree viewer [50]. A sub-selection of 20 species was extracted from the SYMRK orthogroup, and a tree was constructed using the same methods described above. Based on the SYMRK gene models for these 20 species, the splice site at intron 12 for each SYMRK ortholog was added.

### Statistical analysis

Graphs and statistical analysis for mycorrhization quantification were performed using RStudio version 1.1.456. The Ramf R package was used to analyze and display quantitative AM fungal root colonization data [51]. Statistical tests on three classes of mycorrhization efficiency were done using Kruskal–Wallis test in combination with the post-hoc test using Fisher’s least significant difference criterion. Statistical significance was defined as a *p* < 0.01. A statistical test on root growth assays and for nodules number quantification on complementation study was done using a student t-test. Statistical significance for these parameters was defined as a *p* < 0.05.

### Supplementary Information

**Additional file 1.** **Figure S1.** Root architecture of *Trema orientalis* accession RG33 and *Parasponia andersonii* accession WU1 differs. (A) Primary root length 21 days post-germination. (B) Growth of primary root 1-21 days post-germination (mm/day) (C) The average number of lateral roots per plant, and (D) lateral root density (cm-1 main root) 21 days post-germination (E) Average lateral root length in five selected root 21 days post-germination (mm). *n*=5 +/- s.e. Different letters above the bars indicate statistical significance (*p* < 0.05) as determined by student t-test. Tor, red: *T. orientalis* RG33, Pan, blue: *P. andersonii* WU1. Plants were grown in vitro on ½ strength modified Hoagland medium in 12 cm square plates. **Figure S2.** Phylogenetic reconstruction of SYMRK orthologs. Phylogeny was reconstructed on an alignment of SYMRK proteins from 51 plant species belonging to the Nitrogen fixation clade and two species belonging to the Solanales and two species of the Poales. In addition, *Trema* and *Parasponia* SYMRK homologous proteins were added to show that these groups are outside the SYMRK clade. Branch support is indicated by posterior probabilities. Lineages are labelled by species name and gene identifier. **Figure S3.** *Parasponia andersoniisymrk *CRISPR-Cas9 mutant alleles. (A) Structure of *Pansymrk* gene spanning 7,280 bp and possessing 15 exons and 14 introns. Indicated are the positions of two sgRNAs (purple arrowheads) in exons 4 and 5. (B) Sequence alignment of the fourth and fifth exons of *PanSYMRK* in wild type (WT) and the three mutants *Pansymrk-4, Pansymrk-5,* and *Pansymrk-6*. Note:*Pansymrk-4 *is a homozygote mutant possessing a 303 bp deletion whereas line 5 and 6 are bi-allelic. In the bi-allelic mutant lines, both alleles (A and B) are shown. Highlighted in blue and red are the sgRNA target sites and PAM sequences, respectively. **Figure S4.** *Parasponia symrk-5* mutant trans-complementation assay of mycorrhization. (A) Representative image of *Pansymrk-5* *A. rhizogenes* transformed root with empty vector (EV). (B) complementation with *pPanSYMRK:PanSYMRK* gene and (C) trans-complementation with *pPanSYMRK:PanSYMRK*^*GA*^ gene.  Visualization of *Rhizophagus irregularis* infection 6 weeks post-inoculation. **Figure S5.** Analysis of *SYMRK* Intron 12 splice variant expression in *T. orientalis* and *P. andersonii* roots. (A) Mapping of root RNA sequence reads to *SYMRK* gene models of* T. orientalis *and *P. andersonii *showing a ~ 300 bp region around intron 12. (B) Difference in intron retention of *SYMRK* intron 12 and detected by qRT-PCR in* P. andersonii* and *T. orientalis* RG33. The barplot represents the means of three biological replicates ± SD. **Table S1.** Frequency of GA-AG intron splice sites in four plant species. Frequency of GA-AG intron splice sites in annotated gene models of *Parasponia andersonii*, *Trema orientalis*, *Lotus japonicus*, and *Medicago trancatula* genomes. Total number of introns with GA-AG splice sites are based on available annotations. **Table S2.** *T. orientalis* individuals collected in Malaysia, Sabah possess a GA donor splice site at intron 12. Twenty-eight *Trema orientalis* individuals collected at 5 distinct locations in Malaysia, Sabah all possess a non-canonical GA donor splice site at intron 12. *T. orientalis *plants collected from locations outside Malaysia were found to possess a GC donor splice site at intron 12. **Table S3.** List of Golden Gate constructs used in this study. Each construct is identified by a unique number, an ID, and a brief description. The level of Golden Gate assembly for each vector is indicated, as well as the plasmid backbone into which the constructs were cloned. **Table S4.** Primers used in this study. List of primers, their purpose, and their sequence that were used in various applications in this study.

## Data Availability

The datasets analysed during the current study are available in the NCBI SRA repository under BioProject numbers PRJNA272473 and PRJNA272482. Plant material and seeds used in this study can be obtained upon request from the corresponding author.
