# Supplementary material for: A rare non-canonical splice site in Trema orientalis SYMRK does not affect its dual symbiotic functioning in endomycorrhiza and rhizobium nodulation
Source: BMC Plant Biol. 2023 Nov 24;23:587. doi: 10.1186/s12870-023-04594-0 (PMC10668435; doi:10.1186/s12870-023-04594-0)
Supplement: Supplementary file 1 — Additional file 1. Figure S1. Root architecture of Trema orientalis accession RG33 and Parasponia andersonii accession WU1 differs. (A) Primary root length 21 days post-germination. (B) Growth of primary root 1-21 days post-germination (mm/day) (C) The average number of lateral roots per plant, and (D) lateral root density (cm-1 main root) 21 days post-germination (E) Average lateral root length in five selected root 21 days post-germination (mm). n=5 +/- s.e. Different letters above the bars indicate statistical significance (p < 0.05) as determined by student t-test. Tor, red: T. orientalis RG33, Pan, blue: P. andersonii WU1. Plants were grown in vitro on ½ strength modified Hoagland medium in 12 cm square plates. Figure S2. Phylogenetic reconstruction of SYMRK orthologs. Phylogeny was reconstructed on an alignment of SYMRK proteins from 51 plant species belonging to the Nitrogen fixation clade and two species belonging to the Solanales and two species of the Poales. In addition, Trema and Parasponia SYMRK homologous proteins were added to show that these groups are outside the SYMRK clade. Branch support is indicated by posterior probabilities. Lineages are labelled by species name and gene identifier. Figure S3. Parasponia andersoniisymrk CRISPR-Cas9 mutant alleles. (A) Structure of Pansymrk gene spanning 7,280 bp and possessing 15 exons and 14 introns. Indicated are the positions of two sgRNAs (purple arrowheads) in exons 4 and 5. (B) Sequence alignment of the fourth and fifth exons of PanSYMRK in wild type (WT) and the three mutants Pansymrk-4, Pansymrk-5, and Pansymrk-6. Note:Pansymrk-4 is a homozygote mutant possessing a 303 bp deletion whereas line 5 and 6 are bi-allelic. In the bi-allelic mutant lines, both alleles (A and B) are shown. Highlighted in blue and red are the sgRNA target sites and PAM sequences, respectively. Figure S4. Parasponia symrk-5 mutant trans-complementation assay of mycorrhization. (A) Representative image of Pansymrk-5 A. rhizogenes [file 12870_2023_4594_MOESM1_ESM.docx]

**Supplemental figures and tables**


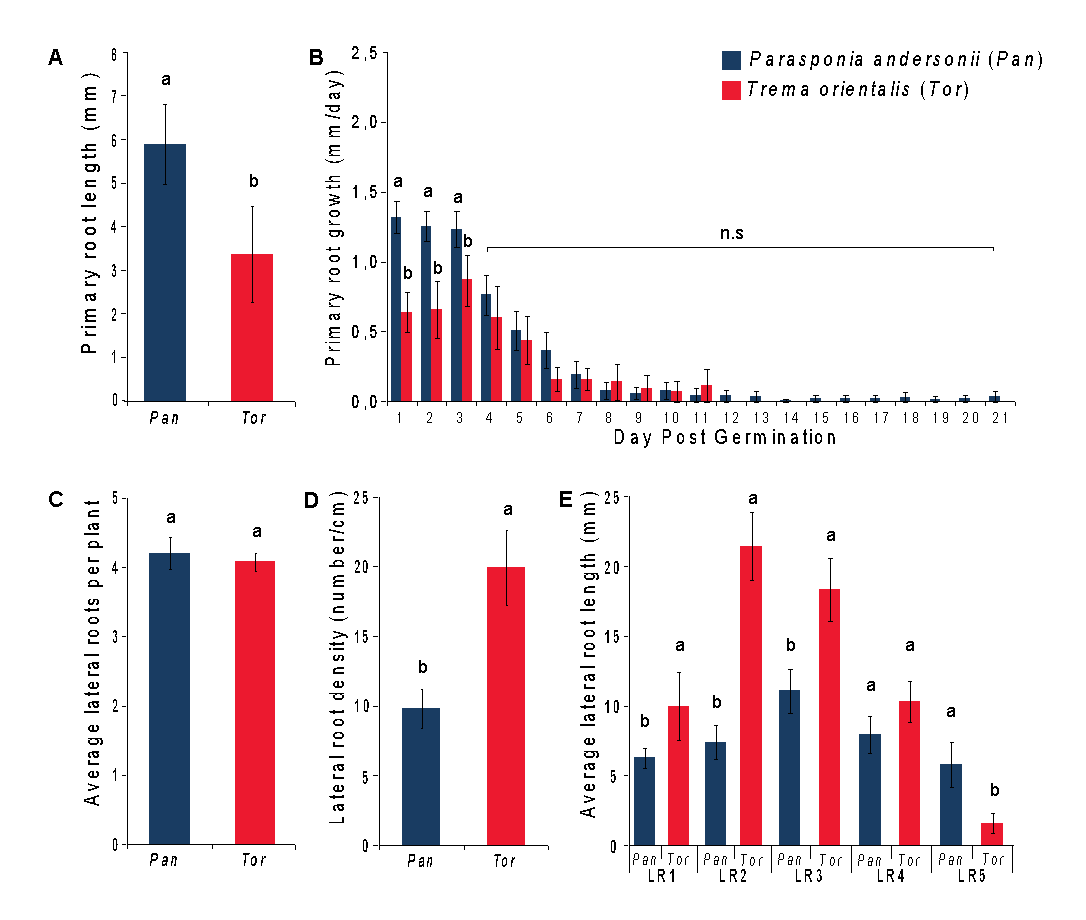


**Figure S1: Root architecture of *Trema orientalis* accession RG33 and *Parasponia andersonii* accession WU1 differs.** (**A**) Primary root length 21 days post-germination. (**B**) Growth of primary root 1-21 days post-germination (mm/day) (**C**) The average number of lateral roots per plant, and (**D**) lateral root density (cm-1 main root) 21 days post-germination (**E**) Average lateral root length in five selected root 21 days post-germination (mm). n=5 +/- s.e. Different letters above the bars indicate statistical significance (p < 0.05) as determined by student t-test. Tor, red: *T. orientalis* RG33, Pan, blue: *P. andersonii* WU1. Plants were grown in vitro on ½ strength modified Hoagland medium in 12 cm square plates.


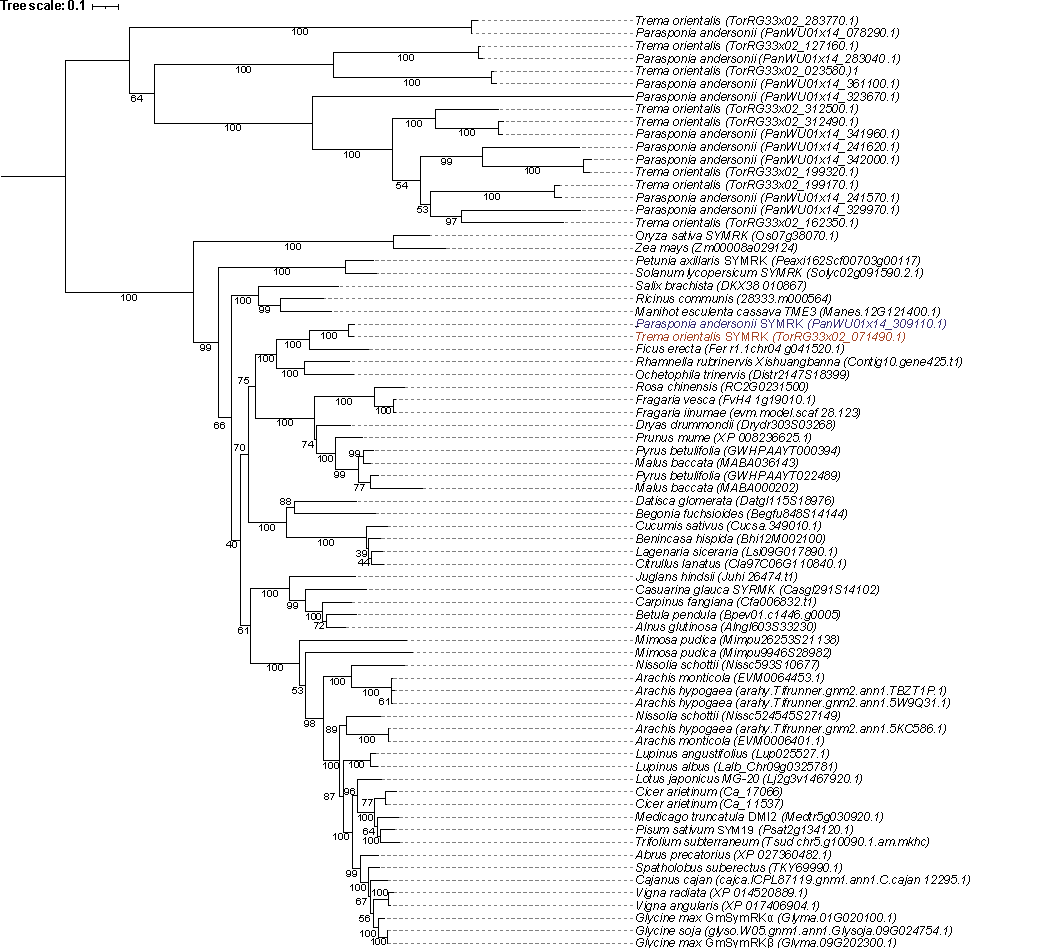


**Figure S2: Phylogenetic reconstruction of SYMRK orthologs.** Phylogeny was reconstructed on an alignment of SYMRK proteins from 51 plant species belonging to the Nitrogen fixation clade and two species belonging to the Solanales and two species of the Poales. In addition, *Trema* and *Parasponia* SYMRK homologous proteins were added to show that these groups are outside the SYMRK clade. Branch support is indicated by posterior probabilities. Lineages are labelled by species name and gene identifier.


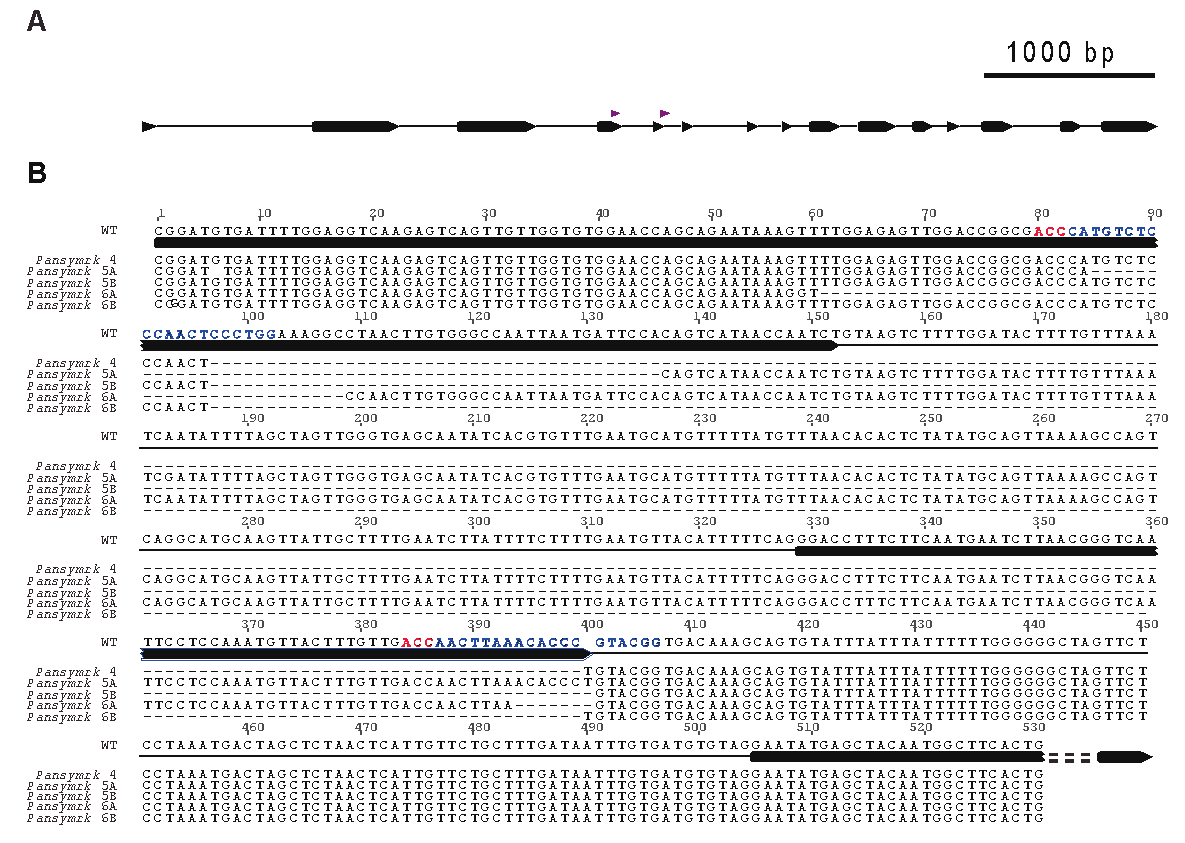


**Figure S3: *Parasponia andersonii* *symrk* CRISPR-Cas9 mutant alleles.** (**A**) Structure of *Pansymrk* gene spanning 7,280 bp and possessing 15 exons and 14 introns. Indicated are the positions of two sgRNAs (purple arrowheads) in exons 4 and 5. (**B**) Sequence alignment of the fourth and fifth exons of *PanSYMRK* in wild type (WT) and the three mutants *Pansymrk-4, Pansymrk-5,* and *Pansymrk-6*. Note: *Pansymrk-4* is a homozygote mutant possessing a 303 bp deletion whereas line 5 and 6 are bi-allelic. In the bi-allelic mutant lines, both alleles (**A** and **B**) are shown. Highlighted in blue and red are the sgRNA target sites and PAM sequences, respectively.


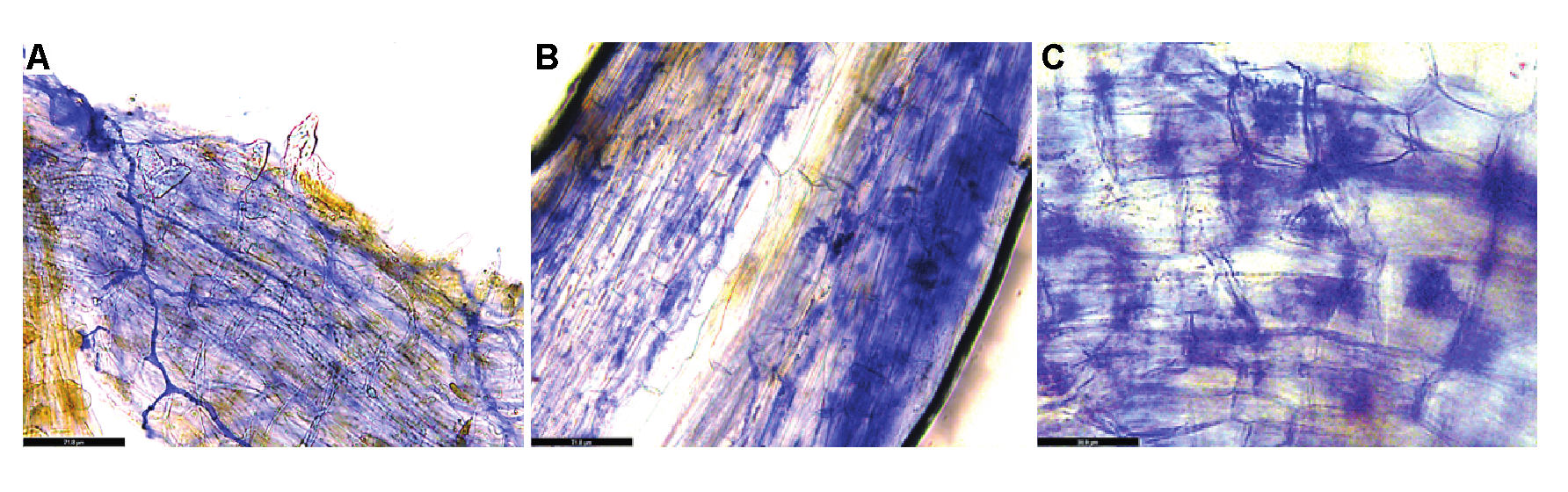


**Figure S4: *Parasponia symrk-5* mutant trans-complementation assay of mycorrhization.** (**A**) Representative image of *Pansymrk-5* *A. rhizogenes* transformed root with empty vector (EV). (**B**) complementation with *pPanSYMRK:PanSYMRK* gene and (**C**) trans-complementation with *pPanSYMRK:PanSYMRK^GA^* gene. Visualization of *Rhizophagus irregularis* infection 6 weeks post-inoculation.


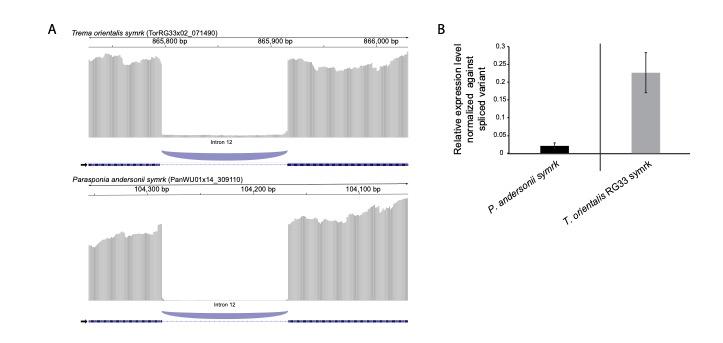
**Figure S5: Analysis of *SYMRK* Intron 12 splice variant expression in *T. orientalis* and *P. andersonii* roots.** (**A**) Mapping of root RNA sequence reads to *SYMRK* gene models of *T. orientalis* and *P. andersonii* showing a ~ 300 bp region around intron 12. (**B**) Difference in intron retention of *SYMRK* intron 12 and detected by qRT-PCR in *P. andersonii* and *T. orientalis* RG33. The barplot represents the means of three biological replicates ± SD.

**Table S1: Frequency of GA-AG intron splice sites in four plant species.**

| **Species + geneiD** | **# introns** | **GA-AG contaning intron** | | **Gene name** | | **Description** |
| --- | --- | --- | --- | --- | --- | --- |
| ***T. orientalis*** |  |  |  | | |  |
| TorRG33x02_013800.1 | 6 | 5 | - | | | ABC transporter ABCE, partial |
| *TorRG33x02_020930.1* | 10 | 1 | - | | | Hypothetical protein |
| TorRG33x02_024300.1 | 3 | 3 | - | | | Hypothetical protein |
| *TorRG33x02_071490.1* | 14 | 12 | - | | | Receptor-like kinase |
| TorRG33x02_098400.1 | 2 | 2 | - | | | Hypothetical protein |
| *TorRG33x02_119330.1* | 1 | 1 | - | | | Ribonuclease |
| TorRG33x02_144520.1 | 2 | 1 | - | | | Hypothetical protein |
| *TorRG33x02_177120.1* | 1 | 1 | - | | | Hypothetical protein |
| TorRG33x02_254520.1 | 9 | 4 | - | | Alpha/Beta hydrolase fold containing protein DNA polymerase III | |
| TorRG33x02_314940.1 | 11 | 5 | - | | | DNA polymerase III, subunit gamma/ tau |
| TorRG33x02_315370.1 | 1 | 1 | - | | | Hypothetical protein |
|  |  |  |  | | |  |
| ***P. andersonii*** |  |  |  | | |  |
| PanWU01x14_022890.1 | 1 | 1 | - | | | Hypothetical protein |
| PanWU01x14_040010.1 | 2 | 1 | - | | | Hypothetical protein |
| PanWU01x14_044300.1 | 1 | 1 | - | | | Hypothetical protein |
| PanWU01x14_108930.1 | 10 | 3 | - | | | 43kDa postsynaptic protein |
| PanWU01x14_144790.1 | 3 | 2 | - | | | Tyrosine-protein kinase |
| PanWU01x14_232680.1 | 7 | 1 | - | | | Zinc finger, C2H2 domain containing protein, partial |
| PanWU01x14_279230.1 | 20 | 8 | - | | | Leucine-tRNA ligase, archaeal |
| PanWU01x14_359300.1 | 8 | 7 | - | | | Carotenoid oxygenase |
|  |  |  |  | | |  |
| ***M. truncatula*** |  |  |  | | |  |
| None |  |  |  | | |  |
|  |  |  |  | | |  |
| ***L. japonicus*** |  |  |  | | |  |
| Lj1g3v4833890.1 | 11 | 10 | - | | | Long chain acyl-CoA synthetase 1-like |
| Lj2g3v0632220.1 | 9 | 9 | - | | | Probable cyclic nucleotide-gated ion channel 20, chloroplastic-like |
| Lj3g3v2661170.1 | 1 | 1 | - | | | Lipoxygenase |
| Lj4g3v0353400.1 | 5 | 2 | - | | | Uncharacterized protein isoform 1 |
| Lj5g3v0837720.1 | 1 | 1 | - | | | F-box/kelch-repeat protein |
| Lj5g3v2045560.1 | 6 | 2 | - | | | Protein tesmin/TSO1-like CXC 2-like |
|  |  |  |  | | |  |
| ***A. thaliana*** |  |  |  | | |  |
| AT1G21750.2 | 9 | 9 | PDI5 | | | PROTEIN DISULFIDE ISOMERASE 5 |
| AT1G62200.1 | 4 | 4 | PTR6 | | | PEPTIDE TRANSPORTER 6 |
| AT1G69020.1 | 10 | 5 | - | | | prolyl oligopeptidase family |
| AT1G73300.1 | 12 | 8 | SCPL2 | | | SERINE CARBOXYPEPTIDASE-LIKE 2 |
| AT2G16960.2 | 17 | 4 | - | | | ARM repeat family |
| AT2G17770.2 | 2 | 1 | FDP | | | FD PARALOG |
| AT3G08900.1 | 3 | 2 | RGP3 | | | REVERSIBLY GLYCOSYLATED POLYPEPTIDE 3 |
| AT3G10350.1 | 10 | 5 | GET3B | | | GUIDED ENTRY OF TAIL-ANCHORED PROTEINS 3B |
| AT3G12520.2 | 17 | 1 | SULTR4;2 | | | SULFATE TRANSPORTER 4;2 |
| AT3G48190.1 | 77 | 73 | ATM | | | TAXIA-TELANGIECTASIA MUTATED 1 |
| AT3G62040.1 | 5 | 2 | - | | | haloacid dehalogenase-like hydrolase family |
| AT4G01800.1 | 19 | 4 | SECA1 | | | CHLOROPLAST SecA |
| AT4G27500.1 | 5 | 5 | PPI1 | | | PROTON PUMP INTERACTOR 1 |
| AT4G35900.1 | 2 | 1 | FD | | | FD |

Frequency of GA-AG intron splice sites in annotated gene models of *Parasponia andersonii*, *Trema orientalis*, *Lotus japonicus*, and *Medicago trancatula* genomes. Total number of introns with GA-AG splice sites are based on available annotations.

**Table S2: *T. orientalis* individuals collected in Malaysia, Sabah possess a GA donor splice site at intron 12.**

| **species** | **accession** | | | **intron 12 donor splice site** | **origin** | **GPS MGRS** | **Latitude & Longitude** | | **altitude (meter)** | |
| --- | --- | --- | --- | --- | --- | --- | --- | --- | --- | --- |
| *T. orientalis* | | RG1 | | CACGAG^GAAAGT | Malaysia, Sabah, Poring | 50NMM 56071 58651 | 5.958664, 116.603094 | | | 1018 |
| *T. orientalis* | | RG2 | | CACGAG^GAAAGT | Malaysia, Sabah, Poring | 50NMM 67379 68352 | 6.046485, 116.705216 | | | 540 |
| *T. orientalis* | | RG4 | | CACGAG^GAAAGT | Malaysia, Sabah, Poring | 50NMM 67379 68352 | 6.046485, 116.705216 | | | 540 |
| *T. orientalis* | | RG5 | | CACGAG^GAAAGT | Malaysia, Sabah, Poring | 50NMM 67494 68312 | 6.046124, 116.706255 | | | 496 |
| *T. orientalis* | | RG8 | | CACGAG^GAAAGT | Malaysia, Sabah, Poring | 50NMM 67137 68377 | 6.046710, 116.703029 | | | 499 |
| *T. orientalis* | | RG9 | | CACGAG^GAAAGT | Malaysia, Sabah, Poring | 50NMM 67137 68377 | 6.046710, 116.703029 | | | 499 |
| *T. orientalis* | | RG16 | | CACGAG^GAAAGT | Malaysia, Sabah, Sayap | 50NMM 51863 83196 | 6.180670, 116.564897 | | | 852 |
| *T. orientalis* | | RG17 | | CACGAG^GAAAGT | Malaysia, Sabah, Sayap | 50NMM 51942 83438 | 6.182860, 116.565609 | | | 716 |
| *T. orientalis* | | RG18 | | CACGAG^GAAAGT | Malaysia, Sabah, Sayap | 50NMM 51951 83446 | 6.182932, 116.565690 | | | 802 |
| *T. orientalis* | | RG19 | | CACGAG^GAAAGT | Malaysia, Sabah, Sayap | 50NMM 51893 82920 | 6.178174, 116.565170 | | | 787 |
| *T. orientalis* | | RG20 | | CACGAG^GAAAGT | Malaysia, Sabah, Sayap | 50NMM 51936 82479 | 6.174185, 116.565562 | | | 835 |
| *T. orientalis* | | RG23 | | CACGAG^GAAAGT | Malaysia, Sabah, Poring | 50NMM 67802 68279 | 6.045827, 116.709038 | | | 467 |
| *T. orientalis* | | RG27 | | CACGAG^GAAAGT | Malaysia, Sabah, Poring | 50NMM 68156 68282 | 6.045856, 116.712237 | | | 445 |
| *T. orientalis* | | RG28 | | CACGAG^GAAAGT | Malaysia, Sabah, Poring | 50NMM 67752 68251 | 6.045573, 116.708587 | | | 461 |
| *T. orientalis* | | RG29 | | CACGAG^GAAAGT | Malaysia, Sabah, Mahua | 50NMM 34597 40705 | 5.796153, 116.409250 | | | 1065 |
| *T. orientalis* | | RG31 | | CACGAG^GAAAGT | Malaysia, Sabah, Mahua | 50NMM 34905 40499 | 5.794292, 116.412034 | | | 1046 |
| ***T. orientalis*** | | | **RG33** | **CACGAG^GAAAGT** | **Malaysia, Sabah, Mahua** | **50NMM 35073 40387** | **5.793281, 116.413552** | | | **1050** |
| *T. orientalis* | | RG34 | | CACGAG^GAAAGT | Malaysia, Sabah, Mahua | 50NMM 36940 38236 | 5.773840, 116.430435 | | | 895 |
| *T. orientalis* | | RG36 | | CACGAG^GAAAGT | Malaysia, Sabah, Mahua | 50NMM 36887 38255 | 5.774012, 116.429956 | | | 887 |
| *T. orientalis* | | RG37 | | CACGAG^GAAAGT | Malaysia, Sabah, Mahua | 50NMM 36703 38442 | 5.775701, 116.428292 | | | 895 |
| *T. orientalis* | | RG38 | | CACGAG^GAAAGT | Malaysia, Sabah, Gunug Alab | 50NMM 27210 43115 | 5.817880, 116.342505 | | | 1827 |
| *T. orientalis* | | RG39 | | CACGAG^GAAAGT | Malaysia, Sabah, Gunug Alab | 50NMM 27221 43132 | 5.818034, 116.342604 | | | 1830 |
| *T. orientalis* | | RG40 | | CACGAG^GAAAGT | Malaysia, Sabah, Gunug Alab | 50NMM 26424 40871 | 5.797573, 116.335429 | | | 1560 |
| *T. orientalis* | | RG41 | | CACGAG^GAAAGT | Malaysia, Sabah, Gunug Alab | 50NMM 27548 38135 | 5.772835, 116.345609 | | | 1340 |
| *T. orientalis* | | RG45 | | CACGAG^GAAAGT | Malaysia, Sabah, Gunug Alab | 50NMM 29782 35934 | 5.752948, 116.365808 | | | 1004 |
| *T. orientalis* | | RG50 | | CACGAG^GAAAGT | Malaysia, Sabah, Crocker Rang, Inobong | 50NMM 03620 49526 | 5.875581, 116.129347 | | | 261 |
| *T. orientalis* | | RG52 | | CACGAG^GAAAGT | Malaysia, Sabah, Crocker Rang, Inobong | 50NMM 03744 50645 | 5.885704, 116.130451 | | | 205 |
| *T. orientalis* | | RG53 | | CACGAG^GAAAGT | Malaysia, Sabah, Crocker Rang, Inobong | 50NMM 03738 50648 | 5.885736, 116.130401 | | | 205 |
| *T. orientalis* | | WU41 | | CACGAG^GCAAGT | China | not determined | not determined | not determined | | |
| *T. orientalis* | | WU42 | | CACGAG^GCAAGT | China | not determined | not determined | not determined | | |
| *T. orientalis* | | WU43 | | CACGAG^GCAAGT | China | not determined | not determined | not determined | | |
| *T. orientalis* | | WU44 | | CACGAG^GCAAGT | China | not determined | not determined | not determined | | |
| *T. orientalis* | | WU45 | | CACGAG^GCAAGT | China | not determined | not determined | not determined | | |
| *T. levigata* | | WU50 | | CACGAG^GCAAGT | China | not determined | not determined | not determined | | |
| *T. tomentosa* | | | WU10 | CACGAG^GCAAGT | Australia | not determined | not determined | not determined | | |
| *P. andersonii* | | | WU1 | CACGAG^GCAAGT | Papua-New Guinea | not determined | not determined | not determined | | |

Twenty-eight *Trema orientalis* individuals collected at 5 distinct locations in Malaysia, Sabah all possess a non-canonical GA donor splice site at intron 12. *T. orientalis* plants collected from locations outside Malaysia were found to possess a GC donor splice site at intron 12.

**Table S3: List of Golden Gate constructs used in this study.**

| **Construct** | | **ID** | **Description** | **Level** | **Backbone** | **contains** |  |
| --- | --- | --- | --- | --- | --- | --- | --- |
| 1 | EC75056 | | *nptII* resistence cassette | 1 | plCH47802 | plCSL70004:*nptII* |  |
| 2 | EC74638 | | 35S_pro_:ΩNLS-Cas9:35S_ter_ | 1 | plCH47742 | plCH41388:35S_pro_ , pAGM5331:ΩNLS, |  |
|  |  |  |  |  |  | plCH41308::aCas9, plCH41414:35S_ter_ |  |
| 3 | EC74578 | | PanSYMRKsgRNA1 | 1 | plCH47761 | plCSL01009:AtU6p, corresponding |  |
|  |  |  |  |  |  | PCR amplicon |  |
| 4 | EC74579 | | PanSYMRKsgRNA2 | 1 | plCH47751 | plCSL01009:AtU6p, corresponding |  |
|  |  |  |  |  |  | PCR amplicon |  |
| 5 | EC74836 | | CRISPR_ctrl | 2 | plCSL4723 | 1R: construct 1, 2F: construct 2, end- |  |
|  |  |  |  |  |  | link plCH41744 |  |
| 6 | EC74796 | | CRISPR_PanSYMRK | 2 | plCSL4723 | 1R: construct 1, 2F: construct 2, 3F: |  |
|  |  |  |  |  |  | construct 3, 4F: construct 4; end-link |  |
|  |  |  |  |  |  | plCH41766 |  |
| 7 | EC75091 | | PanSYMRK Part1 _Pro_:SYMR_gene_: | 1 | pICH47742 | EC75120:SYMRK_Pro,_ EC75123: SYMRK_gene_ part1, EC75124: SYMRK_gene_ |  |
|  |  |  | SYMRK_ter_ |  |  | part2, EC75122: SYMRK_ter_ |  |
| 8 | EC75092 | | PanSYMRK Part1 _Pro_:SYMRK_gene-GA_ | 1 | pICH47742 | EC75120:SYMRK_Pro_, EC75123: SYMRK_gene_ part1, |  |
|  |  |  | :SYMRK_ter_ |  |  | EC75125: SYMRK_gene-GA_ part2, EC75122: SYMRK_ter_ |  |
| 9 | EC75093 | | PanSYMRK Part2_Pro_ | 1 | pICH47732 | EC75121:SYMRK_Pro_ part2 |  |
|  |  |  |  |  |  |  |  |
| 10 | EC75022 | | 35S_Pro_:erGFP_gene_:t35S_ter_ | 1 | pICH47831 | pICH51277:35S_Pro_, EC74047:erGFP_gene_, plCH41414:35S_ter_ |  |
|  |  |  |  |  |  |  |  |
| 11 | EC75220 | | 35S_Pro_:erGFP_gene_:t35S_ter_ | 2 | MOB215_pICS | Dummy1,Dummy2,Dummy3,4R: Construct 10, |  |
|  |  |  |  |  | L4723_modif- | end-link pICH41780 |  |
|  |  |  |  |  | ied to Spec R |  |  |
| 12 | EC75223 | | PanSYMRK_Pro_ Part2, SYMRK_Pro_ Part1, | 2 | MOB215_pICS | 1F: Construct 9, 2F: Construct 7, Dummy 3, 4R: Construct 10, |  |
|  |  |  | SYMRK_gene_:SYMRK_ter_, |  | L4723_modif- | end-link pICH41780 |  |
|  |  |  | 35S_Pro_:erGFP_gene_:35S_ter_ |  | ied to Spec R |  |  |
| 13 | EC75224 | | PanSYMRK_Pro_ Part2, SYMRK_Pro_ Part1, | 2 | MOB215_pICS | 1F: Construct 9, 2F: Construct 8, Dummy 3, 4R: Construct 10, |  |
|  |  |  | SYMRK_gene-GA_:SYMRK_ter_, |  | L4723_modif- | end-link pICH41780 |  |
|  |  |  | 35S_Pro_:erGFP_gene_:35S_ter_ |  | ied to Spec R |  |  |
| 14 | EC75926 | | LjUBQ1_Pro_: PanSYMRK_gene_ part1: | 1 | pICH47811 | EC74013:LjUBQ1_Pro,_ EC75123: SYMRK_gene_ part1, |  |
|  |  |  | tNOS_ter_ |  |  | EC75124: SYMRK_gene_ Part2, pICH41421:tNOS |  |
| 15 | EC75668 | | LjpUBQ1_pro_, PanSYMRK_gene_, | 2 | MOB215_pICS | 1F: Construct 1, 2R:construct 14, Dummy 3, 4R: contstruct 10, |  |
|  |  |  | 35S_Pro_:erGFP_gene_:35Ster |  | L4723_modified to Spec R | end-link pICH41780 |  |
|  |  |  |  |  |  |  |  |

Each construct is identified by a unique number, an ID, and a brief description. The level of Golden Gate assembly for each vector is indicated, as well as the plasmid backbone into which the constructs were cloned.

**Table S4: Primers used in this study.**

| **Name** | **Purpose** | **Sequence** |
| --- | --- | --- |
| PanSYMRK_For | qRT-PCR | GTCCTCGGGTTCCAGTTTG |
| PanSYMRK_Rev | qRT-PCR | ATCACATCCGCATCATTTGG |
| PanEf-1a_For | qRT-PCR | AGACAAGGTTAAGCGTGCAG |
| PanEf-1a_Rev | qRT-PCR | TGCAACTGGGCAACAAACTC |
| PanACT_For | qRT-PCR | CCTCATTGGAATGGAAGCAC |
| PanACT_Rev | qRT-PCR | TTCCAGGAAACATGGTGGAC |
| PanSYMRK_unspliced_For | qRT-PCR | ACAGGAGAAGCATCAGCAAG |
| PanSYMRK_unspliced_Rev | qRT-PCR | ATGAACAAGCTAACATCCAAGG |
| PanSYMRK_spliced_For | qRT-PCR | ACAGGAGAAGCATCAGCAAG |
| PanSYMRK_spliced_Rev | qRT-PCR | ATGTGTCAATCCTCGTGCAG |
| TorSYMRK_unspliced_For | qRT-PCR | CCAACCAGACTTTCGATTGC |
| TorSYMRK_unspliced_Rev | qRT-PCR | CAAGCTAACATCAAAGGCACTG |
| TorSYMRK_spliced_For | qRT-PCR | ACAGGAGAAGCATCAGCAAG |
| TorSYMRK_spliced_Rev | qRT-PCR | ATGTGTCAATCCTCGTGCAG |
| sgRNA-Rv | CRISPR assembly | TGTGGTCTCCAAGCGTAATGCCAACTTTGTAC |
| PanSYMRK_sgRNA1 | CRISPR assembly | TGTGGTCTCAATTGACCCATGTCTCCCAACTCCCGTTTTAGAGCTAGAAATAGCAAG |
| PanSYMRK_sgRNA2 | CRISPR assembly | TGTGGTCTCAATTGACCAACTTAAACACCCTGTAGTTTTAGAGCTAGAAATAGCAAG |
| geno_PanSYMRK-KO-Fw | Genotyping CRISPR mutants | TTCCAGTTTGGGGCCCATTT |
| geno_PanSYMRK-KO-Rv | Genotyping CRISPR mutants | GGAGGAAGAAGGAAAGTCCGG |

List of primers, their purpose, and their sequence that were used in various applications in this study.
